# Hybrid data-driven resilience assessment and enhancement of distribution system for cyclone susceptible zones

**DOI:** 10.1038/s41598-022-13311-0

**Published:** 2022-06-09

**Authors:** Sonal, Debomita Ghosh

**Affiliations:** Birla Institute of Technology Mesra, Ranchi, India

**Keywords:** Electrical and electronic engineering, Climate-change mitigation

## Abstract

The sprawl of distribution system towards the need of smart grid, demands better sustenance and adaptation strategies to deal with high-impact low-frequency (HILF) events. One of the predominant causes of HILF events are natural calamities. Therefore, the resilience assessment of the distribution system is inevitable. The contributions majorly focuses on hybrid data driven approach using micro-phasor measurement unit (μ-PMU), for dynamic voltage, current phasors monitoring, and unmanned aerial vehicle (UAV) confirms structural vulnerability of nodes within network. Mesh grid approach, which analyses cyclone trajectory affecting the network, supplemented identification of most vulnerable part within network. However, priorities of vulnerable nodes are corroborated using complex network (CN) theory. This hybrid data driven approach and spatial parameters are used to estimate appropriate mitigation strategies against HILF scenarios. Hence, resilience analysis based on location parameters and dynamic network conditions are further analyzed based on degree of correlation of location dependent resilience with latitude, elevation, and probable water level. Based on susceptible vulnerable nodes, identification of optimum alleviation schemes is adopted and justified using resilience trapezoid. To validate efficacy of the proposed approach, the analysis is tested on IEEE 33-bus distribution network subjected to 5 cyclone prone geographical coordinates for 20 years cyclone data.

## Introduction

The rapid urbanization, deployment of SCs, and ever-increasing demand of quality power has led to the sprawl of distribution system towards the pre-requisites of smart grid. The distribution system being critical infrastructure of the power system is required to be resilient enough to provide power to the priority customers without interruption^1,2^. Hence, planning of resilient distribution system is significant, also keeping into the perspective of HILF events, caused majorly due to the natural calamities. The impact of cyclones worldwide causes significant damage to infrastructure, with adverse consequences, leading to HILF event^7^. Prompted with the need to cope with the growing cyclone frequencies, sustained power supply in the face of these events require smarter grid-related equipments, that are capable of perceiving real-time system information for monitoring and control^3–5^. In Ref.^6^, power system susceptibility and its planning measures to reduce the impact of extreme wind forces are discussed. In Refs.^8,9^, the quantification of weather severity and its effect on the network is presented in mesh-grid form. Mesh-grid method plays a significant role to transform multi-dimensional HILF event impacting the network, into 2-dimension to analyse the effect of wind trajectory on the network. But, most of the planned management measures are patchy, particularly in the developing nations, due to lack of weather hazard resilient designed networks and non-availability of adequate asset redundancy.

In Ref.^10^, modern technologies such as wide-area monitoring through μ-PMUs are explored to enable faster restoration of the system network. In Ref.^11^, various suggestions and challenges for hardening and operational system restoration models are provided. A range of electrical and topological factors influencing the system’s susceptibility during disasters in a particular region are presented. In Ref.^12^, impacts caused by wind-related events on the distribution system are scrutinized by transforming a large amount of μ-PMU data for feature extraction. The goal is to enhance situational awareness (SA) in the distribution side by monitoring the equipment, distributed energy resources (DERs), and customers being served. In Ref.^13^, voltage and current synchrophasor that are captured by μ-PMUs are used to analyse the damage and assess appropriate restoration techniques. In Ref.^14^, μ-PMU information is co-ordinated with local communication for the post-event restoration of the network. A two-stage problem is formulated to quantify the network parameters. It uses stochastic program for hardening and mixed-integer linear programming method to model the system operability. The sequencing and hardening aspects are unified to form a resilient framework. In Refs.^15–17^, synchronized voltage and current phasors from dispersed locations are considered for pre-storm network assessment. This posed the possibility to improve the power system’s reliability and resiliency, which is presented through a case study. The results show that with μ-PMU, the duration of unplanned outages can be reduced to 51.7%. In Ref.^18^, the pre-event mitigation strategy of reconfiguration is estimated based on the statistical method, with shift and shed load technique using μ-PMU measurements. In Ref.^19^, a kernel principal component analysis (kPCA) is adopted to build the data-driven event detection framework. In Ref.^20^, the greater demand for resilience is achieved by formulating hardening problems into a combinatorial optimization. The operational problems are solved using repeated mixed-integer linear programming formulations and a single crew approximation method. Thus, μ-PMU data provides a structured description of operational parameters. In Refs.^21,22^, the need for optimal hardening and operationally fit distribution networks, resilient to natural disaster is addressed. Though the μ-PMU data provides SA and considers inaccuracies in system-related parameters, but measurement of network parameters with only μ-PMU is unable to provide the component damage status. The physical bending of components like distribution poles and damage of overhead lines are difficult to monitor and analyse for post-disruption management. So, a method is required for monitoring the status of components during such events.

In Ref.^23^, the application of aerial systems for disaster recovery and relief activities in the power sector are explored. UAV has been promising device for meteorological measurements. In Ref.^24^, pole infrastructure damage assessment through UAV-based inspection of line video is detailed. In Refs.^25,26^, for cyclone Harvey, a case study is illustrated, where SA is improved by collecting and analysing quantitative datas. These datas provide SA for extreme event response and also for maintenance purposes. In Ref.^27^, method of optical imaging is used as the primary data for power line inspection and deep learning technique for data analysis. However, the lack of training data is its main challenge. In Refs.^28,29^, automatic pole angles analysis by deep learning technique and computer vision method is determined. In Ref.^27^, the resilience of pole by the application of big data technology for fast image recognition of unhealthy towers is researched. In Ref.^30^, aerial remote sensing based on UAV is operated alongside wireless systems. This increased the number of users to establish a line-of-sight links (LOS) for communication. In Refs.^31,32^, artificial vision technique for the exploitation of information provided from different sensors are applied. Although, the use of UAV ensures investigation of damaged poles and lines, but it cannot provide the information about the interconnection of physical infrastructure and smart technologies. Both μ-PMU and UAV are individually utilized for damage assessment of distribution network during HILF events. However, integration of both the technologies guarantee fast, time-synchronized data monitoring and control of both physical infrastructure and network operation, at the same time.

In Ref.^33^, the concept of CN as a set of connected nodes representing real interconnected systems is explained. In Refs.^34–36^, the CN concept for power grids is demonstrated, in which generators and loads are considered as nodes. It aids to identify central components with the maximum influence on the desired system performance. Both μ-PMU data and UAV can collect information but, have drawbacks in providing prioritising the vulnerable nodes of network, which is a gap for further research. The identification of vital nodes is important for decision-making by the network operators aiming for a desired level of system performance. Therefore, combining CN with μ-PMU data and UAV can be an effective strategy in increasing resilience by first identifying the vulnerable points of the network for node prioritization. Along with it, the mesh-grid approach aids in improving the visualization owing to wind-related events by examining the relatively better controllable portion of a network.

Different resilience enhancement measures are effective for different locations as per the geographic and topological aspects. Also, the loss of resilience is not always linearly associated with network parameters, but is associated with the location aspects i.e., latitude, elevation, and water level. In Ref.^37^, the mitigation measures of power system due to abnormal events ranging from minor to major outages are discussed. The planning alternatives of different systems for resilience and its related indices are compared. In Ref.^40^, the degree of association between topological features and their expected resilience under extreme weather conditions are estimated by using the Pearson correlations. In Refs.^41,42^, the correlation between wind speed and load, for planning and reliability analysis are discussed. However, the correlation or dependency analysis has not been applied for relating the extent of impact of weather-related uncertainties on network recovery. Therefore, a data-driven approach for finding the association between the network’s features and location-dependent resilience indices must be explored. In addition, location-dependent correlation can have a significant impact for the best possible recovery response to wind-related events. The major objectives of the present research are therefore as follows:

1. Mesh-grid approach to transform the multi-dimension impact of cyclone trajectory on the distribution network to 2-dimensional form for appropriate identification of vulnerable part within the network.
2. Hybrid data driven approach based on μ-PMU and UAV aided monitoring, combined with CN, to identify and prioritize the vulnerable nodes within the distribution network.
3. Estimation of degree of correlation hinged on topographic conditions of node bringing out the importance of latitude, elevation, and probable water level, which may encumber the resilience of distribution system planning.
4. Identification of optimum mitigation strategies for enhancement of network resilience based on minimum, intermediate, and maximum cyclone trajectory and its verification using resilience trapezoid.
5. Testing the potency of the proposed methodology using IEEE 33-bus distribution system subjugated to five cyclone susceptible geographical emplacement (18.7669° S, 46.8691° E), (27.717573° N, − 80.40344° W), (14.519780° N, 120.98969° E), (51.561705° N, 0.829468° W), and (− 25.33223° S, 113.8864° E) based on historical 20 years cyclone data.

The second section presents the brief review on resilient planning and operation of distribution systems. It proposes method for resilience planning and operation using hybrid data-driven approach to identify the optimum mitigation strategy and enhance the network resilience during cyclone. The third section analyses the case study and results. The last section concludes the research.

## Brief review on resilient planning and operation of distribution systems

The expansion of SCs, increasing demand of power, and growing urbanization challenges are the need for resilient planning of the smart distribution systems. Increased frequency of extreme events has motivated to study the detailed methodology and effectiveness of resilient planning and operation of distribution systems.

### Mesh-grid representation to estimate the impact of cyclone on network

**Figure 1:**
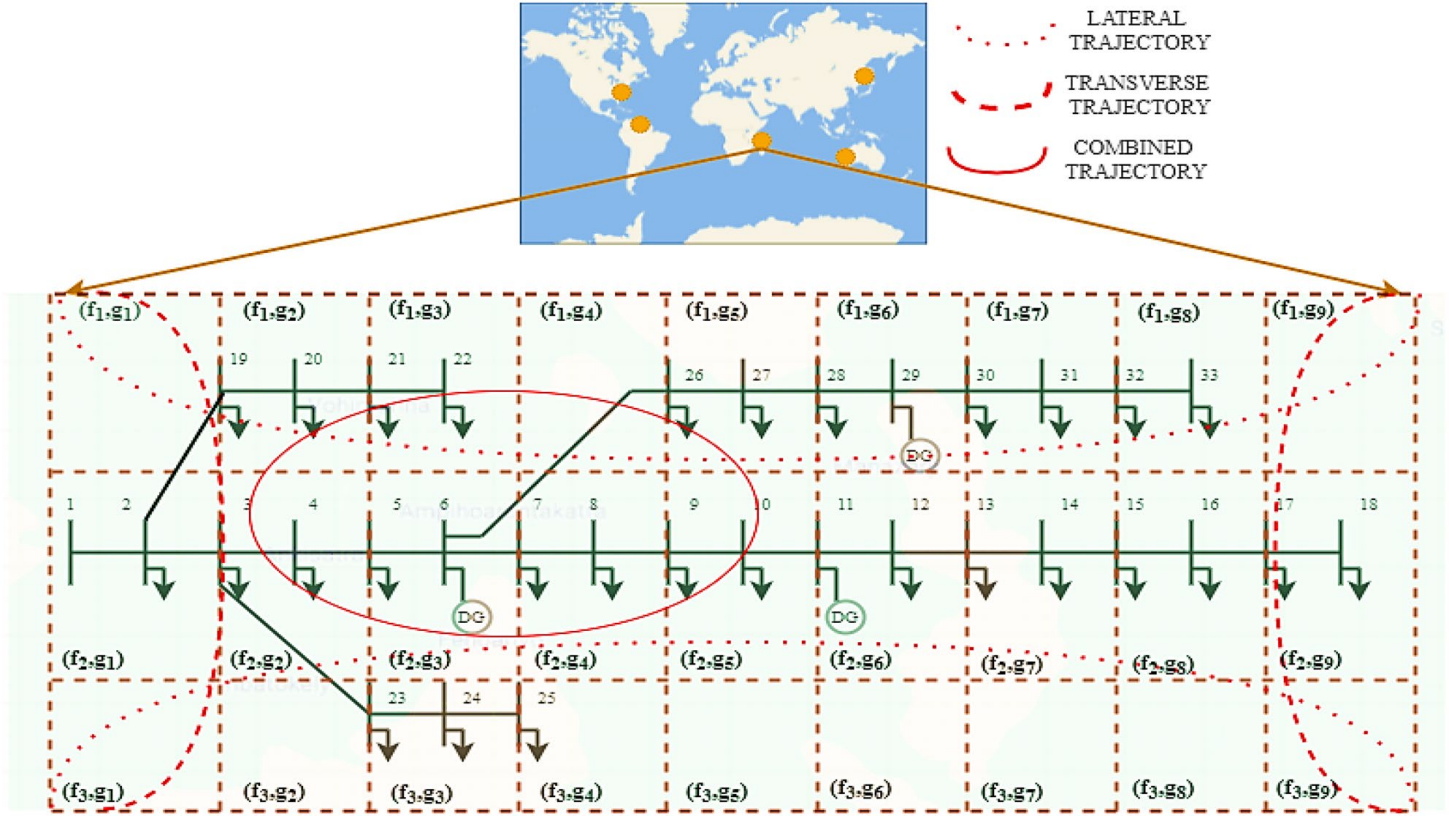
Cyclone trajectory extrapolation on mesh-grid for IEEE 33-bus network.

The mesh-grid approach depicts the interaction of the cyclone with network, considering location variation which can be framed as two-dimensional representation^41,42^. This approach can be applied across any region with differing trajectories, as per the historical data of the site. This transformation into two-dimension aids in efficient modelling and rendering of muti-dimensional cyclone track impact on the network operation. The superposition of geographical location with physical network can be employed to find the impact of line outages in extreme events^14,54^. The lateral, transverse, and combined cyclone trajectories on network, is shown in Fig. 1. Here, the IEEE 33-bus system is illustrated in a mesh-grid with $f$ rows and $g$ columns, represented as $f\times\ g$ cells. Each cell on the mesh can be represented by its coordinates such as cell ${({f}}_{1},{{g}}_{1})$. The lateral, transverse, and combination of trajectories are used to interpret the network outages as per the paths of cyclones. The portion lying on the trajectory plotted may trip with a high probability.

The duration of time for which a location is exposed to wind storm depends mainly on its size and speed. Algebraic relations between speed ${V}_{m}$, coefficient *n*, height of the location, $Z$ and latitude $\varphi$, are derived by fitting power-law expression of sites, as in Eqs. (1) and (2) respectively^10^.

1 $${V}_{m}=2.20 (\sqrt{Z})$$

2 $$n=0.431+0.136{V}_{m}-0.006 \varphi$$

Outside the eye of cyclone, the wind decays exponentially, that is estimated by regression relation, ${Y}_{1}$ as in Eq. (3)^14^.

3 $${Y}_{1}=270.5-4.78 {V}_{m}+6.17 \varphi$$

For cyclone trajectory, the radius of maximum wind speed, ${r}_{max}$ concept is used. The parameterized formula for calculating ${r}_{max}$ according to the maximum wind speed, ${V}_{m}$ and the latitude, $\varphi$ is as in Eq. (4)^10^.

4 $${r}_{max}=46.4 \mathrm{exp}(-0.01555 {V}_{m}+0.0169 \varphi )$$

The deployment of SCs require intricate planning owing to increase in demand. At these hubs of urbanization, the distribution side caters to satisfy the increasing demands of the customers. The mesh-grid representation of IEEE 33-bus distribution network is superimposed at these hubs to analyse the impact of system operation due to geographical features. The same network is placed at all five major cyclone prone zones. The same concept can be applied to any network. According to world meteorological organization, five major tropical cyclone formation zones of the world identified are South-west Indian Ocean, Arabian Sea and Bay of Bengal, North Pacific, North Atlantic and North-east Pacific, and South-west Pacific and South-east Indian Ocean region^40^. Based on 20 years of cyclone data of these 5 zones, 5 most cyclone-prone regions are selected with coordinates [(18.7669° S, 46.8691° E), (27.717573° N, − 80.40344° W), (14.519780° N, 120.98969° E), (51.561705° N, 0.829468° W), and (− 25.33223° S, 113.8864° E)], which are used for analysis in the subsequent sections.

### Micro-PMU based network dynamism monitoring

μ-PMUs provide real-time voltage and current data with high accuracy. It confers network observability considering the minimal number of required μ-PMUs to monitor overall system status. The number of μ-PMUs providing maximum observability of network, $\mathit{max}O\left(\mathcal{L}\right)$ is given as in Eq. (5).^7,43,45^

5 $$\mathit{max}O\left(\mathcal{L}\right)= \sum_{j\epsilon L}{w}_{j}{\mathcal{L}}_{j}, \mathrm{with}, {\mathcal{L}}_{j}\epsilon \left\{\mathrm{0,1}\right\},$$

where, ${w}_{j}$ represents relative weight of bus *j*, ${\mathcal{L}}_{j}$ represents the μ-PMUs status. Value 1 denotes presence of μ-PMU at bus *j*, else 0, means μ-PMUs are not installed at that particular bus.

μ-PMUs are capable of precisely measuring time synchronized data leading to enhanced SA of the network. The overall network monitoring using data obtained by the μ-PMUs check the bus voltage ${V}^{j}$ and branch current ${I}^{jk}$ for each node *j* of a distribution system, as in Eqs. (6) and (7)^6^.

6 $${V}_{min}^{j}\le \left|{V}^{j}\right|\le {V}_{max}^{j}$$

7 $${I}^{jk}\le {C}^{j}$$

Due to the HILF event, some lines get disrupted and are needed to be repaired. The μ-PMU shows status of nodes connected to it. To check the interconnection of buses by distribution lines Eq. (8) is used. In this case, signals received at the bays of μ-PMU determines if buses *i* and *j* are connected or not.

8 $${\iota }_{ij}=\left\{\mathrm{0,1}\right\}, \forall (i,j)\epsilon \overline{\iota }$$

HILF events lead to degraded power quality. During such events, voltage sags are the most common power quality disturbance usually associated with network disturbances. The actual voltage at node *j*, ${V}^{j}$ is rechecked to observe if its value lies within a range set by rated voltage ${V}_{rated}$ and the corresponding tolerance $\varepsilon$ as given by Eq. (9) from the observable μ-PMUs.

9 $${V}_{rated}-\varepsilon {V}_{rated}\le {V}^{j}\le {V}_{rated}+\varepsilon {V}_{rated}$$

The changes of the nodal voltages ${\Delta V}_{j}$ and branch currents ${\Delta I}_{jk}$ due to the HILF event is analysed. It is represented in terms of voltage and branch current before the event ${V}_{j}^{BE}$ and ${I}_{Jk}^{BE}$, and after the event ${V}_{j}^{AE}$ and ${I}_{jk}^{AE}$, respectively, as shown in Eqs. (10) and (11).

10 $${\Delta V}_{j}={V}_{j}^{AE}-{V}_{J}^{BE}$$

11 $${\Delta I}_{jk}={I}_{jk}^{AE}-{I}_{Jk}^{BE}$$

Thus, though the μ-PMU data provides enhanced SA and identifies the inaccuracies in system related parameters, but the physical bending of components like distribution poles and damage of overhead lines are difficult to monitor. The pole tilt and line disruption are not observable by μ-PMUs, so a method is required for monitoring the status of components during such HILF events.

### UAV based location dependent structural surveillance of the network

**Figure 2:**
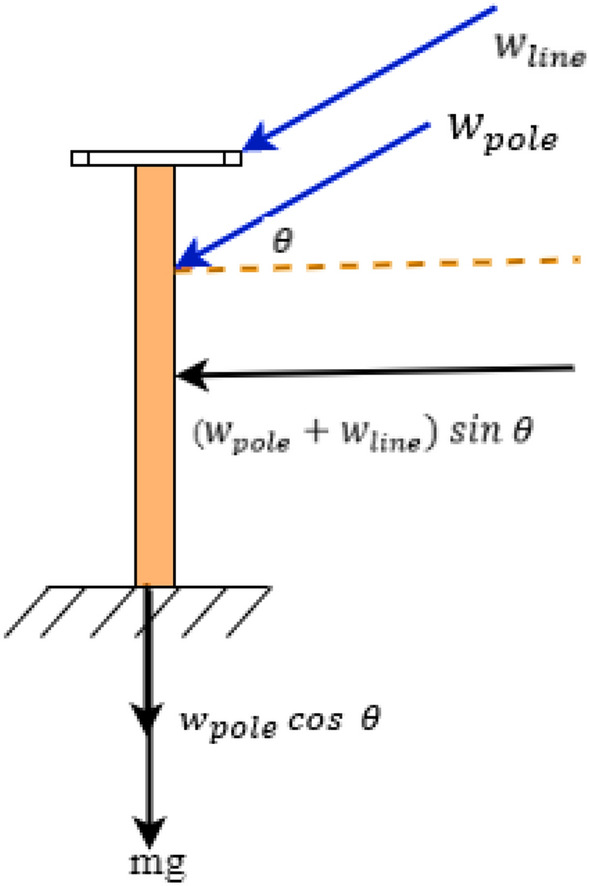
Free body diagram of poles and lines under the influence of wind event.

The UAVs are equipped with global positioning system (GPS), that can record location information of unhealthy components for timely repair. Images depict the impact of wind storm on electric distribution systems. Free body diagram of poles and lines under the influence of wind event is represented in Fig. 2. It depicts the three major types of forces acting on poles and lines, that are gravitational force, wind force on pole and wind force on overhead lines.

From Fig. 2, the force on overhead lines and poles and their respective areas are considered for evaluation of stress. If the image and GPS details obtained from UAV has ‘*np*’ number of poles that are shaking or vibrating, then based on Eq. (12) stress on poles, $\varsigma$ can be estimated.

12 $$\varsigma = \frac{{w}_{pole} cos \theta }{{\mathrm{\rm A}}_{pole}}+\frac{{w}_{line} cos \theta }{{\mathrm{\rm A}}_{line}}$$

The moment of a pole, ${\mathrm{\rm M}}_{pole}$ depends on component of gravitational force ${\mathrm{\rm M}}_{g}^{\perp }$, component of wind force on pole, ${\mathrm{\rm M}}_{w}^{\perp }$ , and component of wind force on overhead line, ${\mathrm{\rm M}}_{ln}^{\perp }$ , as obtained by Eq. (13).

13 $${\mathrm{\rm M}}_{pole}={\mathrm{\rm M}}_{g}^{\perp }+{\mathrm{\rm M}}_{w}^{\perp }+{\mathrm{\rm M}}_{ln}^{\perp }=\left\{\left({F}_{g}^{\perp }\times {d}_{g}\right)+\left({F}_{w}^{\perp }\times {d}_{w}\right)+\left({F}_{ln}\times {d}_{ln}\right)\right\}$$

${\mathrm{\rm M}}_{pole}$ is used to assess the pole rupture stress ${\varsigma }_{rupture}$, represented in terms of its diameter *dia*, as shown in Eq. (14).

14 $${\varsigma }_{rupture}= \frac{32 {\mathrm{\rm M}}_{pole} }{\pi {dia}^{3}}$$

Based on the values of ${\mathrm{\rm M}}_{pole},$ the infrastructural resilience can thus be classified based on the health of poles as given by Eq. (15)^21^.

15 $$if{\mathrm{\rm M}}_{pole} =\left\{\begin{array}{c}\zeta \le 55.16\times {10}^{6}N/{m}^{2} - resilient\\ 55.16\times {10}^{6}<\zeta <70.26\times {10}^{6}N/{m}^{2}- moderately resilient\\ 70.26\times {10}^{6}N/{m}^{2}\le \zeta -non resilient\end{array}\right.$$

To analyse the effect of cyclonic storm on lines, firstly the line can be represented as first-order polynomial ${y}_{l}^{j}$, with slope $a$ and intercept *b*, given in Eq. (16)^27^.

16 $${y}_{l}^{j}=a{x}_{l}^{j}+b$$

Also, availability of both the left as well as right sides of images for the affected distribution lines during the inspection is required. Usually, 100 pixels from the left as well as right sides of the distribution line are captured instead of the entire image. The slope of distribution lines, ${\Theta }_{l}$ is measured in terms pole heights ${h}_{l}^{1}$ and ${h}_{l}^{2}$, with ${\mathrm{\rm Z}}_{l}^{\mathrm{1,2}}$ as the span between them, as shown in Eq. (17)^27^.

17 $${\Theta }_{l}=arctan\left(\frac{{h}_{l}^{1}-{h}_{l}^{2}}{{\mathrm{\rm Z}}_{l}^{\mathrm{1,2}}}\right)$$

This approach is convenient and logical because the lines appear straight in inspection images. The narrow field of view of the overhead lines by the UAV camera conforms to the general catenary curve, $C$ shown as in equation, for, $\alpha =1, {C}^{2}=1, dx=1 and dy=\mathrm{sinh}(t)$, respectively^28^.

18 $$\frac{{y_{l}^{2\alpha j} }}{{1 + y_{l}^{{\prime}2} }} = \frac{{y_{l}^{2\alpha j} dx^{2} }}{{dx^{2} + dy^{2} }} = C^{2}$$

If the horizontal length in a recorded, image is approximately 3.5 m for an 8° horizontal angle of view, when the UAV camera is 25 m away from the distribution lines, then the ${\Theta }_{l}$ measured should lie within the ranges as shown in Eq. (19)^23^.

19 $$-15^\circ <{\Theta }_{l}<15^\circ$$

The UAVs especially when the μ-PMUs fail can assist in enhancing resilience by collecting images of damaged lines and poles during the wind storm. In this inspection, images depend on the specifications of the UAV camera and also the distance between the camera and the distribution lines. Utilizing UAV-based imagery for storm damage collection is significant. However, the ability to monitor physical pole bending does not provide overall information of all storm related operational malfunction within the network. Thus, this methodology when integrated with μ-PMU data provides better observation into any malfunction induced due to cyclone. Further prioritizing strategy of affected nodes within a network to enhance resilience is detailed in the next section.

### Hybrid μ-PMU, UAV and CN based approach for network node prioritization strategy 

The detailed methodology and the effectiveness of data driven approach using μ-PMU and UAV for resilience estimation of the distribution system by CN are presented in this section. Historical cyclone data are used to estimate cyclone trajectory based on the mesh-grid structure. The corresponding dynamism of network parameters at each node and aerial surveillance is monitored using μ-PMU and UAV. The implementation of CN is examined based on the topographic conditions of the nodes (buses) and connectivity of edges (distribution lines).

The CN-based resilience framework ranks critical components on a priority basis, ${\aleph }_{rank}$ with respect to node capacity ${\mathbb{N}}_{c}$, node centrality degree ${D}_{n}$, and connectivity impact *CI*. This assists in carrying out timely recovery of network damage condition owing to a severe cyclonic storm as in Eq. (20)^9,54^.

20 $${\aleph }_{rank}=\left\{\begin{array}{c}if{\mathbb{N}}_{c}=\left(1+\delta \right) {l}_{c}=1, and \\ {D}_{n} :\sum_{j=1}^{{n}_{t}}{p}_{de}=1, and\\ CI :1-\frac{{\mathrm{\rm T}}_{a}}{{n}_{t}}=1\end{array}\right.$$

Slow moving cyclones may take long hours to move past a particular location. This may lead to extreme winds and rain that lasts up to 12 h. It can be time consuming to randomly select location that will be impacted in the system due to cyclone. Therefore, node ranking based on priority using CN approach on mesh-grid representation is used to study the cyclone trajectory. It prioritizes the nodes of a distribution network based on CN parameters and accordingly analyse the vulnerable points. The nodes previously unobservable by μ-PMUs are considered to be re-ranked using UAV by application of CN based parameters. The implementation of node prioritization using hybrid μ-PMU, UAV and CN based approach is presented in Algorithm 1. In this, node prioritization of network, assessing the topographic conditions of the nodes (buses) and connectivity of edges (distribution lines) are analysed to re-rank the unobservable nodes.
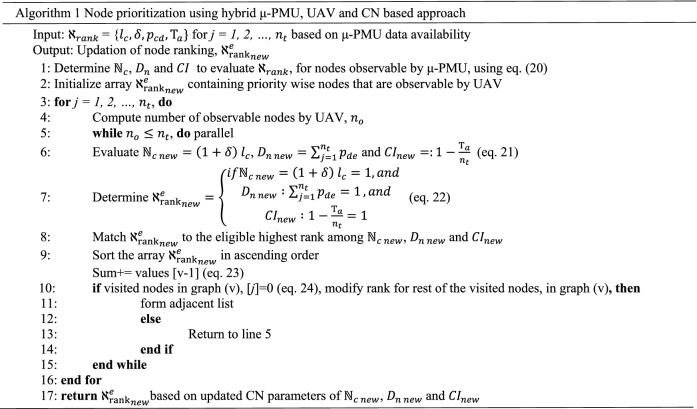


Thus, node ranking using hybrid μ-PMU, UAV and CN of distribution system provides suggestions on node-wise priority classification. It is a useful method to set out network connectivity benchmarks during HILF events.

### Resilience enhancement using location-dependent correlation analysis for network mitigation strategy 

**Figure 3:**
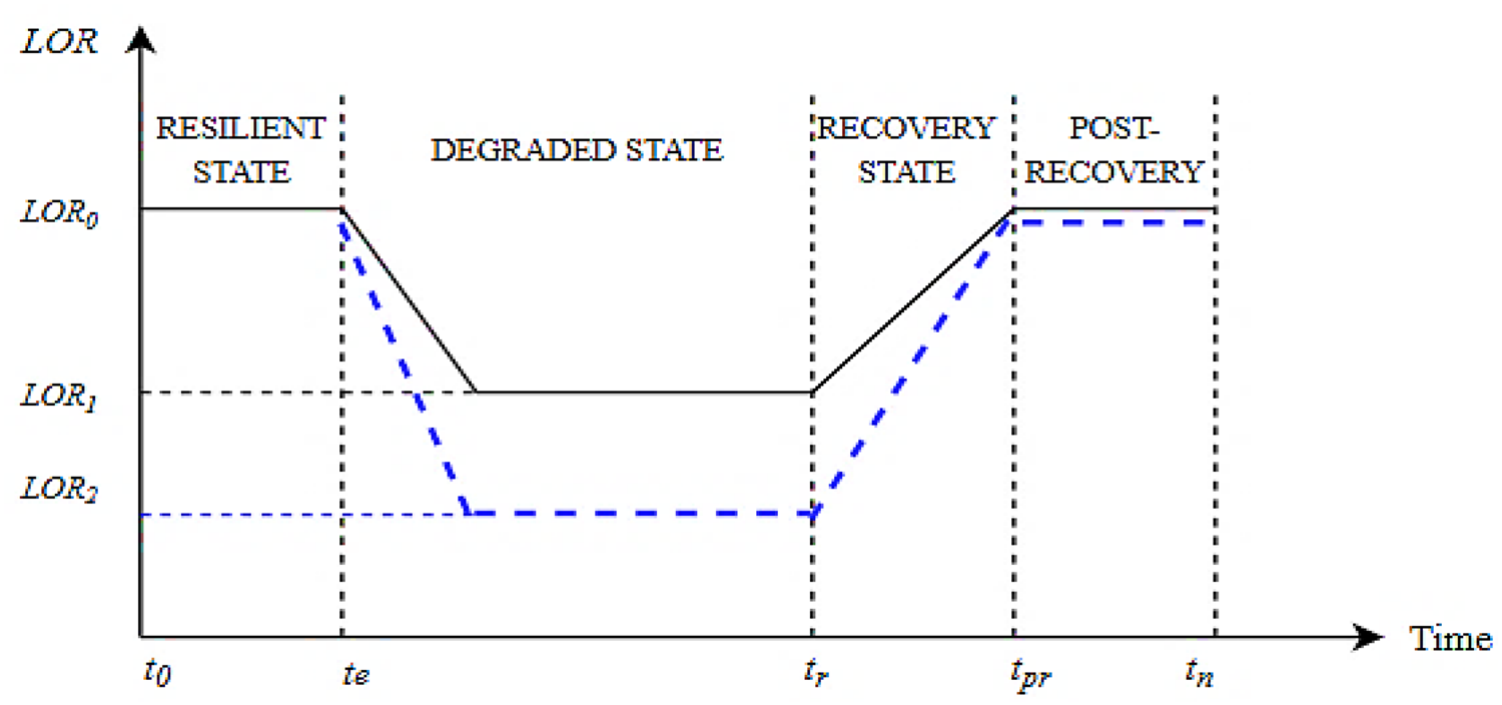
Resilience trapezoid for different level of resilience (LOR).

The location dependent analysis helps to compare network resilience under similar cyclonic conditions and varied geographic location. Correlation between the location parameters i.e., latitude, mean elevation, and probable water level are utilized to find out the most appropriate mitigation strategy for a network located at 5 cyclone prone geographical coordinates based on 20 years of cyclone data. So, this estimation helps in streamlining adequate network mitigation techniques for different regions under a similar set of events. To describe distribution system performance characteristics in terms of resilience metrics due to cyclone, the resilience trapezoid curve is used^38^. The representation of the level of resilience (LOR) is shown in Fig. 3.

The normal level of performance is denoted by ${LOR}_{0}$. ${t}_{e}$ is the time at which wind related event impacts the network. Thus, the network performance changes from ${LOR}_{0}$ to ${LOR}_{1}$ in the degraded state and ${LOR}_{1}$ to ${LOR}_{2}$ if appropriate mitigation strategy is not provided on time. The recovery capabilities of a network are limited by the available restoration resources. Therefore, the recovery rate and duration of resilience trapezoid curve would be different. The use of appropriate mitigation technique is initiated at ${t}_{r}$. After this, the network returns to almost its original performance level. The resilience of system ${\mathcal{R}}_{i}\left(\tau \right)$, at any location *i,* with respect to time $\tau$, is obtained using Eq. (21)^39^.

21 $${\mathcal{R}}_{i}\left(\tau \right)=\frac{\underset{\tau ={t}_{0}}{\overset{{t}_{n}}{\int }}{LOR}_{i}\left(\tau \right)d\tau }{\underset{\tau ={t}_{0}}{\overset{{t}_{n}}{\int }}{LOR}_{0}d\tau }$$

Here, $${LOR}_{0}=1>{LOR}_{1}> {LOR}_{2}$$

The cyclonic regions are classified based on latitude, longitude, elevation, and probable water level features because these parameters vary with varied geographical location. The location-dependent resilience variation with latitude, elevation, and probable water level represented by ${cr}_{\mathcal{R},\varphi }, {cr}_{\mathcal{R},\mathcal{\hslash }}, {\mathrm{and} cr}_{\mathcal{R},\varpi }$, respectively, forms the resilience metrics, as given by Eq. (22).

22 $$\mathcal{R}={\{cr}_{\mathcal{R},\varphi }, {cr}_{\mathcal{R},\mathcal{\hslash }}, {cr}_{\mathcal{R},\varpi }\}$$

The correlation between the location parameters of latitude φ, or mean elevation $\hslash$, or of probable water level $\varpi$ with expected resilience aspect $\mathcal{R}$, with parameters of reconfiguration, splitting, and pole reinforcement, can be expressed as given in Eqs. (23)–(25)^7^.

23 $${cr}_{\mathcal{R},\varphi }=\frac{\sum_{c=1}^{m}(\varphi -\overline{\varphi })(\mathcal{R}-\overline{\mathcal{R} })}{\sqrt{\sum_{c=1}^{m}{(\varphi -\overline{\varphi })}^{2}{(\mathcal{R}-\overline{\mathcal{R} })}^{2}}} or c{r}_{\mathcal{R},\mathcal{\hslash }}=\frac{\sum_{c=1}^{m}(\hslash -\overline{\hslash })(\mathcal{R}-\overline{\mathcal{R} })}{\sqrt{\sum_{c=1}^{m}{(\hslash -\overline{\hslash })}^{2}{(\mathcal{R}-\overline{\mathcal{R} })}^{2}}}, \mathrm{or} {cr}_{\mathcal{R},\varpi }=\frac{\sum_{c=1}^{m}(\varpi -\overline{\varpi })(\mathcal{R}-\overline{\mathcal{R} })}{\sqrt{\sum_{c=1}^{m}{(\varpi -\overline{\varpi })}^{2}{(\mathcal{R}-\overline{\mathcal{R} })}^{2}}}$$

where,

24 $$\overline{\varphi }=\frac{{\varphi }_{1}+{\varphi }_{2}+{\varphi }_{3}+\dots +{\varphi }_{m}}{m}, \overline{\mathcal{R} }=\frac{{\mathcal{R}}_{1}+{\mathcal{R}}_{2}+{\mathcal{R}}_{3}+\dots +{\mathcal{R}}_{m}}{m}, \overline{\hslash }=\frac{{\hslash }_{1}+{\hslash }_{2}+{\hslash }_{3}+\dots +{\hslash }_{m}}{m} and \overline{\varpi }=\frac{{\varpi }_{1}+{,\varpi }_{2}+{\varpi }_{3}+\dots +{\varpi }_{m}}{m},$$

25 $$-1\le p\le 1$$

Using correlation analysis of ${cr}_{\mathcal{R},\varphi }, {cr}_{\mathcal{R},\mathcal{\hslash }}, {cr}_{\mathcal{R},\varpi }$, it is observed that the degree of correlation, *p* value varies from − 1 to + 1 as reflected in Eq. (25). There is no evidence of correlation if ${cr}_{\mathcal{R},\varphi }, {cr}_{\mathcal{R},\mathcal{\hslash }}, {cr}_{\mathcal{R},\varpi }$, is a value close to 0. A positive association of linear correlation exists if ${r}_{\mathcal{R},\varphi }, {r}_{\mathcal{R},\mathcal{\hslash }}, {r}_{\mathcal{R},\varpi }$ is positive and is closer to 1, and a negative linear correlation exists if ${cr}_{\mathcal{R},\varphi }, {cr}_{\mathcal{R},\mathcal{\hslash }}, {cr}_{\mathcal{R},\varpi }$, is negative and is closer to -1. These ${cr}_{\mathcal{R},\varphi }, {cr}_{\mathcal{R},\mathcal{\hslash }}, {cr}_{\mathcal{R},\varpi }$, values are used to check whether the resilience planning is suitable and the type of resilience-based planning necessary for each scenario. If *p* > *0.5*, then the value is statistically significant. If *p* < *0.5*, then the value is weakly correlated. The correlation values of latitude, longitude, elevation, and probable water level features with the expected resilience level of a region is estimated. These parameters vary with varied geographical location so, with the failure of nodes within the cyclone trajectory and the degree of dependency varies.

**Figure 4:**
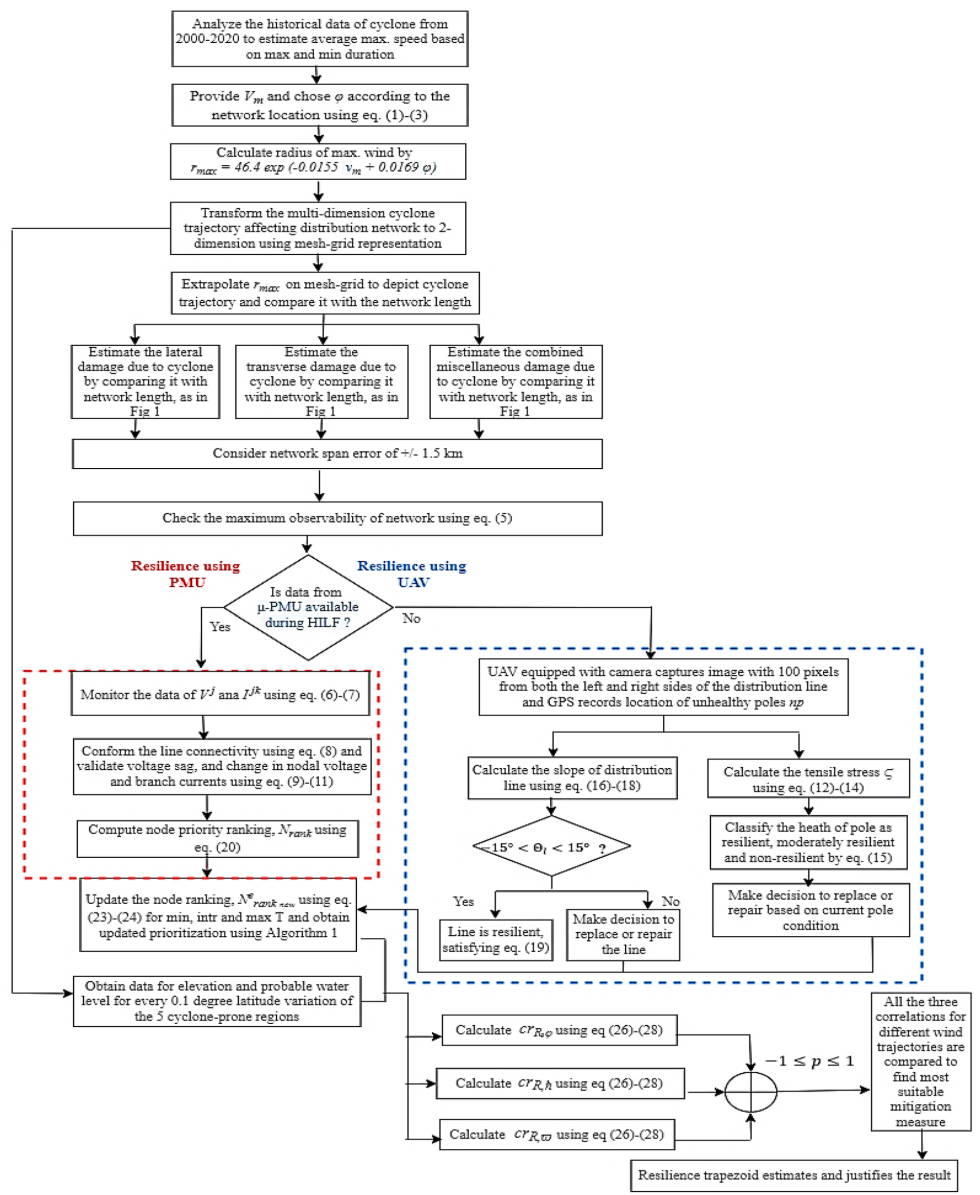
Proposed flowchart for hybrid data-driven resilience assessment and enhancement of distribution system.

Figure 4 represents the proposed flowchart for resilience assessment and enhancement using hybrid data driven approach. The impact of cyclone trajectory on network is represented as 2-dimension using mesh-grid approach. For analysis due to impact of cyclone on the network, μ-PMUs and UAV assisted monitoring is done. Further, to prioritize the nodes according to its importance, CN is also integrated. Resilience based on complex network and correlation value sets benchmark to update the node-wise prioritization of load capabilities during HILF events. Therefore, the location-dependent updating of node includes the geographical location of the network, cyclone intensity, and the properties of node capacity ${\mathbb{N}}_{c}$, node centrality degree ${D}_{n}$, and connectivity impact $CI$. For estimating appropriate mitigation measures reflecting location dependency, the concept of correlation is applied. It assesses the resilience of network with respect to location parameters of latitude, longitude, elevation, and probable water level. The value of positive, negative or no correlation, with respect to trajectory gives the suitable mitigation strategy to be adopted. Finally, resilience trapezoid is used to depict the enhancement of network resilience with suitable mitigation strategies, thus leading to a self-sustained distribution system.

## Analysis of results and discussion

In this section, distribution system planning based on location-dependent structural resilience parameters by utilizing μ-PMU and UAV is applied on the IEEE 33-bus system. The optimal DG size are 913.05 kW, 882.86 kW, and 1079.05 kW at bus no. 12, 24, and 30 respectively^14,54^. The μ-PMUs are installed at the buses. Several literatures are available for μ-PMU location determination based on multiple constraints^43–53^. The optimal μ-PMU locations at the buses, with maximum weight, as per the connectivity are 32, 11, 13, 15, 18 and 31, for overall network observability^43,45^.

For the case study of location dependent resilience, 5 cyclone prone geographical co-ordinates (18.7669° S, 46.8691° E), (27.717573° N, − 80.40344° W), (14.519780° N, 120.98969° E), (51.561705° N, 0.829468° W), and (− 25.33223° S, 113.8864° E) of America, Africa, Asia, Europe, and Australia are chosen. So, considering the length and width of the 33-bus network, at every 0.1° latitude variation, the elevation, and probable water level are taken. Using mesh-grid representation as in Fig. 1, the 2-dimension transformation considering 0.1° = 11.1 km are taken at every 3 km^2^ area. This mesh-grid cell granularity is sufficient to capture the change in latitude, elevation, and probable water level of a distribution system.

The worldwide representation of average windspeed during cyclones and number of samples considered per year for sustained windspeed of 10-min, 3-min, and less than 3-min data are collected for the year 2000 to 2020. Based on the historical data for 21 years, the average wind speed (in kmph) is compiled, for each latitude considered. These datas are beneficial for finding out the ${r}_{max}$ range using Eqs. (1)–(4), and are compared with the network length. The ${r}_{max}$ affecting the line disruption of IEEE 33-bus network are estimated by extrapolation. Based on the respective range of ${r}_{max}$, the trajectory wise disturbances are classified as minimum, intermediate, and maximum. It indicates the distribution system, line disruption on mesh-grid enclosed within the radius of maximum wind speed. The computation of ${r}_{max}$ leading to corresponding results for cyclone trajectories of min T, intr T, and max T, wise line disruption, are summarized in Table 1. The location with higher possibility of ${r}_{max}$ tends to have a greater number of line disruptions due to larger part of the network being enclosed within the range of ${r}_{max}$, as observed with the case of cyclone region 5. Similarly, cyclone region 1 has lower ${r}_{max}$, leading to a smaller number of lines being disrupted because a smaller part of the network lies within the range of ${r}_{max}$.

**Table 1:** Results based on mesh-grid to determine the probable trajectory of cyclone affecting ieee 33-bus system for 5 cyclone prone regions.

Cyclone region	Co-ordinates	No. of samples/year	Avg. windspeed range (kmph)	Avg. ${r}_{max}$ range (km)	Cyclone trajectory wise line disruption		
					Min T	Intr T	Max T
1	18.76691° S, 46.86913° E	49	(205.3, 207.4)	(1.14, 1.34)	16–17	2–19	27–31, 7–11
2	27.71757° N, − 80.4034° W	42	(238.5, 240.5)	(1.58, 1.76)	16–17	2–19	28–33, 8–13
3	14.519780° N, 120.9897° E	50	(240.1, 242.1)	(1.21, 1.40)	16–17	19–20	27–30, 7–10
4	51.561705° N, 0.82947° W	29	(195.4, 197.4)	(1.17, 1.42)	16–17	19–20	27–31, 7–11
5	− 25.33223° S, 113.8864° E	23	(161.2, 163.3)	(4.96, 5.18)	13–14	12–13	5–6, 26–33, 14–15

To validate the effect of cyclone trajectories leading to possible disruptions, the network needs to be monitored. The cyclone trajectories are hence referred to find out the corresponding vulnerable node, firstly based on only μ-PMU and then by only UAV. The trajectory wise network parameters of ${V}^{j}$, and ${I}^{jk}$ for all the nodes are calculated. For analysing the observation based on only μ-PMU, ${V}^{j}$, and ${I}^{jk}$ are calculated for each node of IEEE 33-bus system using Eqs. (6)–(7). The nodes which are falling within the range of ${r}_{max}$, for min T, intr T, and max T, shows a value of zero as there are no outputs due to the failure of multiple nodes, and loss of observability during HILF events. The distribution line operational parameter limits are checked using Eqs. (9)–(11), and results for network disturbance output using only μ-PMU are tabulated in Table 2, under the column of network disturbance output using only μ-PMU. Again, application of only UAV based status tracking of distribution line and poles are presented in Table 2, under the column of line slope estimation, ${\Theta }_{l}$ and trajectory wise stress, ${\varsigma }_{j}$, respectively. In such case, pole and distribution line fault diagnosis is performed conveniently for difficult to reach locations by capturing images. For computing the output using only UAV, ${\varsigma }_{rupture}$ is calculated using Eqs. (12)–(14), that is considered for classification of ${\mathrm{\rm M}}_{pole}$ using Eq. (15). For min T, intr T, and max T. The distribution line slope ${\Theta }_{l}$ exceeding the normal range of ± 15°, is determined using Eqs. (16)–(18). This reflects candidate location for line disruption, with values of 15.03° (line connecting bus 15 and 16) and 15.05° (line connecting bus 16 and 17) for min T. 15.36° (line connecting bus 17 and 18) and 15.73° (line connecting bus 18 and 19) for intr T. 15.3693° (line connecting bus 27 and 28) and 15.62289° (line connecting bus 28 and 29) for max T. It highlights that the distribution lines between two towers having large altitude differences lead to a larger range of ${\Theta }_{l}$ values. The nodes satisfying both the conditions of ${V}^{j}$, and ${I}^{jk}$ datas being available, in addition to line slope estimation of $-15^\circ <{\Theta }_{l}<15^\circ$, combined with $\varsigma$ $\le 55.16\times {10}^{6}N/{m}^{2}$, are considered to be resilient network parameters (RNP). The range for moderately resilient network parameters (MRNP) are $55.16\times {10}^{6}<\zeta <70.26\times {10}^{6}N/{m}^{2}$. Else, the violation of operational parameters results in μ-PMU based potentially unsafe network, with stress $70.26\times {10}^{6}N/{m}^{2}\le \zeta$, leading to non-resilient network parameters (NRNP). For determining the resilience of a network using only μ-PMU and only UAV for IEEE 33-bus network, subjected to region 1 are detailed, and is presented in Table 2. For rest four regions, similar observations are obtained.

**Table 2:** Vulnerable node identification using only μ-PMU and only UAV based on cyclone trajectory for region 1.

Node		Network disturbance output using only μ-PMU						Structural resilience output using only UAV						Observation based on only μ-PMU or only UAV output
		Trajectory wise voltage, $${V}^{j}$$ (p.u.)			Trajectory wise current, $${I}^{jk}$$ (A)			Line slope estimation, $${\Theta }_{l}$$ (°)			Trajectory wise stress, $${\varsigma }_{j}$$($${10}^{6}N/{m}^{2})$$			
*j*	*k*	Min T	Intr T	Max T	Min T	Intr T	Max T	Min T	Intr T	Max T	Min T	Intr T	Max T	
1	2	1.0000	1.0000	1.0000	5.46	5.46	5.66	0.126	0.043	0.158	15.2	15.0	43.1	RNP
2	3	0.9975	0.9976	0.9986	5.31	5.31	5.31	0.091	0.157	0.238	16.5	16.8	21.3	RNP
3	4	0.9857	0.9853	0.9930	4.43	4.43	4.46	0.130	0.007	0.078	15.1	23.9	51.9	RNP
4	5	0.9795	0.9788	0.9914	6.44	6.44	6.52	0.180	0.017	0.130	17.8	31.1	17.6	RNP
5	6	0.9735	0.9724	0.9901	2.98	2.97	3.03	0.107	0.212	0.079	18.5	30.1	40.1	RNP
6	7	0.9579	0.9559	0.9874	2.76	2.76	2.85	0.095	0.129	0.079	30.0	24.0	55.0	RNP
7	8	0.9553	0.9529	0.9868	9.74	9.72	10.1	0.241	0.221	15.62	31.0	43.1	69.7	MRNP
8	9	0.9518	0.9488	0.0000	9.71	9.67	0.00	0.077	0.154	15.73	10.9	20.1	84.1	UAV based NRNP
9	10	0.9475	0.9433	0.0000	2.73	2.72	0.00	0.130	0.077	0.180	10.8	15.1	65.9	UAV based NRNP
10	11	0.9437	0.9384	0.0000	2.72	2.71	0.00	0.050	0.119	0.134	11.5	28.7	86.7	UAV based NRNP
11	12	0.9431	0.9376	0.0000	2.33	2.31	0.00	0.119	0.164	0.022	13.8	43.2	65.8	μ-PMU based NRNP
12	13	0.9422	0.9363	0.0000	2.98	2.97	0.00	0.174	0.228	0.130	14.7	50.2	43.0	μ-PMU based NRNP
13	14	0.9387	0.9312	0.0000	2.97	2.95	0.00	0.138	0.131	0.106	43.1	39	50.0	μ-PMU based NRNP
14	15	0.9375	0.9293	0.0000	6.17	6.11	0.00	0.052	0.149	0.141	47.8	44.7	20.0	μ-PMU based NRNP
15	16	0.9370	0.9281	0.0000	2.60	2.57	0.00	15.03	0.093	0.083	55.8	43.1	25.9	μ-PMU based NRNP
16	17	0.9367	0.9269	0.0000	2.70	2.67	0.00	15.05	0.018	0.101	59.6	47.4	21.3	μ-PMU based NRNP
17	18	0.0000	0.9252	0.0000	0.00	2.67	0.00	0.145	15.36	0.124	75	53.1	51.5	UAV based NRNP
18	19	0.0000	0.9247	0.0000	0.00	4.15	0.00	0.079	15.73	0.130	67.1	65.2	70.2	UAV based NRNP
19	20	0.9970	0.0000	0.9981	4.48	0.00	4.48	0.104	0.075	0.109	20.9	81	43.7	UAV based NRNP
20	21	0.9935	0.0000	0.9946	4.46	0.00	4.47	0.131	0.128	0.0428	20.5	65.9	53.3	μ-PMU based NRNP
21	22	0.9928	0.0000	0.9939	4.46	0.00	4.46	0.25	0.119	0.106	19.6	44.5	46.5	μ-PMU based NRNP
22	23	0.9922	0.0000	0.9933	4.46	0.00	4.66	0.238	0.028	0.058	21.4	51.7	10.8	μ-PMU based NRNP
23	24	0.9823	0.9820	0.9895	4.61	4.61	4.65	0.089	0.15	0.073	22.5	57.5	32.1	RNP
24	25	0.9760	0.9757	0.9832	20.7	20.7	20.9	0.172	0.092	0.182	23.6	46.7	38.2	RNP
25	26	0.9729	0.9726	0.9801	20.6	20.6	20.8	0.101	0.225	0.183	23.1	23.7	12.8	RNP
26	27	0.9563	0.9543	0.9872	2.83	2.83	2.93	0.050	0.115	0.156	26.1	30.7	70.2	RNP
27	28	0.9541	0.9521	0.9871	2.83	2.82	2.93	0.107	0.173	15.36	24.7	29.9	64.2	MRNP
28	29	0.9443	0.9423	0.0000	2.72	2.72	0.00	0.116	0.081	15.62	26.3	33.9	83.1	UAV based NRNP
29	30	0.9369	0.9349	0.0000	5.94	5.92	0.00	0.084	0.113	0.175	28.6	31.6	63.4	UAV based NRNP
30	31	0.9338	0.9319	0.0000	26.9	26.9	0.00	0.151	0.221	0.032	28.4	43.4	77.1	UAV based NRNP
31	32	0.9303	0.9283	0.0000	7.02	7.01	0.00	0.091	0.111	0.103	27.5	42.9	57.9	μ-PMU based NRNP
32	33	0.9295	0.9276	0.0000	9.86	9.84	0.00	0.150	0.132	0.250	29.5	33.3	50.2	μ-PMU based NRNP
33	–	0.9293	0.9273	0.0000	3.06	3.05	0.00	–	–	–	31.4	31.8	46.4	μ-PMU based NRNP

From the results summarized in Table 2, it is observed that distribution lines are non-resilient for wind force exerted on pole at an angle of 0°, moderately-resilient for 15–45°, and resilient for 60–90° angle respectively. However, the application of either μ-PMU or UAV alone is not sufficient to provide the needful information and identify the priority nodes for required mitigation strategies to be adopted for enhancing the network resilience. Combining CN with μ-PMU data and UAV pinpoints the prioritized vulnerable nodes of the network for applying appropriate mitigation measures.

Table 3 thus summarizes the results of hybrid data driven approach for cyclone trajectories in region 1 (18.7669°S, 46.8691°E). It considers the potentially unsafe network condition obtained from the observation of Table 2, and ranks the vulnerable nodes for min, intr and max T by hybrid data driven approach. The column in Table 3 for ranking based on μ-PMU data extraction uses the CN-based framework to rank the critical components on a priority basis. Using Eq. (20), ${\aleph }_{rank}$ is calculated with respect to ${\mathbb{N}}_{c}$, ${D}_{n}$, and *CI*. For μ-PMU, UAV and CN oriented node ranking as in Table 3, node ranking is considered for all those nodes that were unobservable by μ-PMU. By UAV the unobservable nodes are monitored, and CN is applied to re-rank the nodes for each trajectory to obtain ${{\aleph }_{\mathrm{rank}}^{e}}_{new}$, using Algorithm 1 and Eq. (24). It is observed that if the affected node have a comparatively high ranking, then it impacts the operation of the distribution system to a larger extent. These high-risk points of the network are further considered for correlation analysis with the location aspects. Similarly, for all other cyclone prone regions, the hybrid data driven ranking of nodes are computed, based on network impact as per min, intr and max T of cyclone.

**Table 3:** Results of hybrid data driven approach based vulnerable node Prioritization for cyclone trajectories in region 1 (18.7669°S, 46.8691°E).

Node		Ranking based on μ-PMU data extraction			μ-PMU, UAV and CN oriented node ranking		
*j*	*k*	Trajectory wise node ranking, ${\aleph }_{rank}$ = {${\mathbb{N}}_{c}, {D}_{n}, CI\}$			Trajectory wise node ranking, ${{\mathrm{\aleph }}_{\mathrm{rank}}^{e}}_{new}$ = {${\mathbb{N}}_{c new}, {D}_{n new}, {CI}_{new}\}$		
		Min T	Intr T	Max T	Min T	Intr T	Max T
1	2	1	1	1	1	1	1
2	3	2	2	2	2	2	2
3	4	3	3	3	3	3	3
4	5	5	5	5	5	5	5
5	6	11	8	11	11	8	11
6	7	4	4	4	4	4	4
7	8	12	9	12	12	9	12
8	9	13	10	–	13	10	17
9	10	14	11	–	14	11	18
10	11	15	12	–	15	12	19
11	12	23	20	–	23	20	25
12	13	24	21	–	24	21	26
13	14	25	22	–	25	22	27
14	15	26	23	–	26	23	28
15	16	27	24	–	27	24	29
16	17	28	25	–	28	25	30
17	18	–	26	–	29	26	31
18	19	–	28	–	31	28	32
19	20	6	–	6	6	31	6
20	21	9	–	9	9	33	9
21	22	10	–	10	10	32	10
22	23	30	–	16	32	30	16
23	24	7	6	7	7	6	7
24	25	8	7	8	8	7	8
25	26	29	27	15	30	27	15
26	27	16	13	13	17	13	13
27	28	17	14	14	17	14	14
28	29	18	15	–	18	15	20
29	30	19	16	–	19	16	21
30	31	20	17	–	20	17	22
31	32	21	18	–	21	18	23
32	33	22	19	–	22	19	24
33	–	31	29	–	33	29	33

The ecological indicators of latitude, mean elevation, and probable water level are mathematical illustration of physical surface. The distribution system’s complexity, exposure, and geographic reach results in inherently greater vulnerability due to HILF events. Latitudes help to analyse temperature gradient of a location affecting wind trajectory. Historical data for HILF disturbances highlight the impact of alteration in geographical aspect of latitude and mean elevation. The potential intensity of cyclone at higher mean elevation leads to widespread disturbances to the structural part of network by increasing the failure probability. Hydrological feature of probable water level indicates the probability of inundation and failure of distribution system components during cyclone. These non-climatic aspects impact extent of exposure to cyclones by curtailing resilience of the network. This study helps in proper decision making by providing an insightful understanding of a region’s distribution infrastructure planning and operation. It allows decision makers and electric operators to understand which location-based features should be focused to improve the performance of that region’s electric supply and therefore achieve a better overall resilience during cyclones.

Table 4 summarizes the result for degree of correlation for location dependent resilience, $\mathcal{R}$ and the possible mitigation strategies for min, intr and max T in the 5 cyclone regions. Using Eqs. (23)–(25), the correlation factors ${cr}_{\mathcal{R},\varphi }, {cr}_{\mathcal{R},\mathcal{\hslash }}, {cr}_{\mathcal{R},\varpi }$ with expected resilience aspect $\mathcal{R}$, through parameters of reconfiguration, splitting, and pole reinforcement, are evaluated. The trajectory has strong dependence on the network connectivity, and correlation with location aspects indicate its significance of the extent of mitigation viability. For elevation & reconfiguration, *p* value is 0.17 for min T, 0.99 for intr T, and -0,24 for max T, for region 1. This implies that reconfiguration is less significant for min T, highly significant for intr T, while not desirable for max T case. This is due to the absence of the main branch in the network topology for min T and intrt T, where the majority power is flowing. The disruption of main branch leads to loss of connection between the lateral branches, making the entire segment unavailable. Thus, the probability of a network losing the main component that is responsible for serving priority customers becomes high. The correlation value of probable water level and reconfiguration has *p* value of 0.38 for min T, 0.46 for intr T, and 0.27 for max T case. This suggests it can be weakly viable for minimum and intermediate trajectory of cyclone disturbance.

**Table 4:** Identification of the degree of correlation of location dependent resilience metrics and possible mitigation strategies for minimum, intermediate, and maximum cyclone trajectories.

S.no	Factors of correlation	Trajectory wise correlation	*p* value				
			Region1	Region 2	Region 3	Region 4	Region 5
1	Elevation and reconfiguration	Min T	0.17	0.55	0.30	0.22	0.78
		Intr T	0.99	0.99	0.99	0.96	0.99
		Max T	− 0.24	− 0.5	− 0.19	− 0.5	− 0.02
2	Probable water level and reconfiguration	Min T	0.38	0.04	0.33	0.33	0.49
		Intr T	0.46	0.73	0.82	0.99	0.99
		Max T	0.27	0.26	− 0.13	− 0.57	− 0.84
3	Elevation and splitting	Min T	0.11	0.37	0.04	0.47	0.61
		Intr T	0.99	0.98	0.99	0.97	0.99
		Max T	0.45	0.01	0.39	0.38	0.37
4	Probable water level and splitting	Min T	− 0.98	− 0.42	− 0.22	− 0.53	− 0.75
		Intr T	0.28	0.87	0.88	0.95	0.98
		Max T	0.65	− 0.02	0.42	0.15	0.39
5	Probable water level and pole reinforcement	Min T	− 0.08	− 0.50	− 0.02	− 0.37	− 0.33
		Intr T	0.82	0.94	0.95	0.99	0.97
		Max T	0.89	0.90	0.56	0.78	− 0.15

For the second mitigation factor of splitting, its correlation with elevation for intr T has a high correlation value of 0.99, which can be considered as a good option. It is less impactful for splitting strategy for min T with *p* 0.11. The uncertainty between the connected nodes is responsible for low correlation values. However, for max T, the value of 0.45 can be considered as a weak option. For splitting and probable water level, *p* value is − 0.98 for min T, 0.28 for intr T, and 0.65 for max T, for region 1. This implies that splitting is less significant for intr T, highly significant for max T, while not desirable for min T case. This is due to a smaller number of components impacted by water logging impact, therefore showing a weak positive correlation. Probable water level tends to increase the probability of inundation leading to increased chances of component failure.

For the third mitigation factor of pole reinforcement, the *p* value with probable water level is 0.82 and 0.89 for intr T and max T, respectively. The correlation between probable water level and pole reinforcement describes the effect of water level on pole failure and repair dynamics using pole reinforcement changes. It is impactful for intr T and max T cases having a significantly positive correlation of 0.82 and 0.89 respectively. However, min T has negative effect with *p* value of -0.08. This factor should be analysed with cost perspective due to the additional cost and resources required for pole reinforcement alteration. These positive values reflect the system’s readiness towards handling the HILF scenarios with an average loss of performance. Similar to region1, the results for other cyclone prone regions are analysed.

Table 5 summarizes the results for distribution system LOR for the most probable cyclone prone regions with different mitigation measures. It shows the results for LOR-oriented mitigation strategies based on different phases of *pd*, *dp*, *dg*, *rp* and *pres* presented through resilience trapezoid. Based on cyclone trajectory, resilience trapezoid graph for mitigation strategies of network i.e., reconfiguration, splitting, and pole reinforcement are assessed. For the first cyclone prone region (18.76691° S, 46.86913° E), without any mitigation technique, it is observed that any failure will immensely impact the functionality of components within the average ${r}_{max}$ range of disturbance, as per LOR using Eq. (25). The resilience level drops to 0.34 during *dp*. The *rp* is 0.633, 0.655, and 0.756, for splitting and reinforcement scenarios. The *pres* value is 0.753 and 0.811, for reconfiguration and splitting, respectively. This partially restored state denotes that their quota of restoration depends on the technical constraints but reinforcement prevents further inclination or debasement of the poles. Pole reinforcement strategy during planning stage will not allow the system to succumb to degraded state. However, if temporary reinforcement is provided as a mitigation measure, then LOR is improved to reach to *res* of 0.894. The strategy of reconfiguration and splitting takes a shorter period to mitigate and also has a higher rate of recovery than the pole reinforcement technique. This is because it isolates the damaged components within the ${r}_{max}$ and restores the power supply quickly in that area to serve the priority customers. Also, the opening and closing of tie switches and sectionalizing require even less time. For both splitting and reconfiguration, DG back up is required to be provided to endorse power related issues. If optimum mitigation strategy of network, i.e., reconfiguration, splitting, and pole reinforcement are applied on time then the system regains its performance level to 0.753, 0.811, and 0.894, respectively.

**Table 5:** Resilience assessment and enhancement using optimum mitigation strategy in cyclone prone regions.

Cyclone prone region	Mitigation strategy 1: network reconfiguration					Mitigation strategy 2: network splitting					Mitigation strategy 3: pole reinforcement				
	Resilience trapezoid phases					Resilience trapezoid phases					Resilience trapezoid phases				
	pd	dp	dg	rp	pres	pd	dp	dg	rp	pres	pd	dp	dg	rp	res
1	1	0.184	0.475	0.633	0.753	1	0.204	0.655	0.702	0.811	1	0.226	0.736	0.756	0.894
2	1	0.671	0.361	0.557	0.699	1	0.745	0.498	0.561	0.616	1	0.822	0.559	0.542	0.679
3	1	0.680	0.455	0.601	0.756	1	0.755	0.627	0.617	0.776	1	0.834	0.704	0.625	0.856
4	1	0.541	0.448	0.592	0.740	1	0.599	0.618	0.645	0.766	1	0.662	0.695	0.639	0.844
5	1	0.711	0.123	0.190	0.578	1	0.789	0.170	0.210	0.501	1	0.871	0.191	0.232	0.600

**Figure 5:**
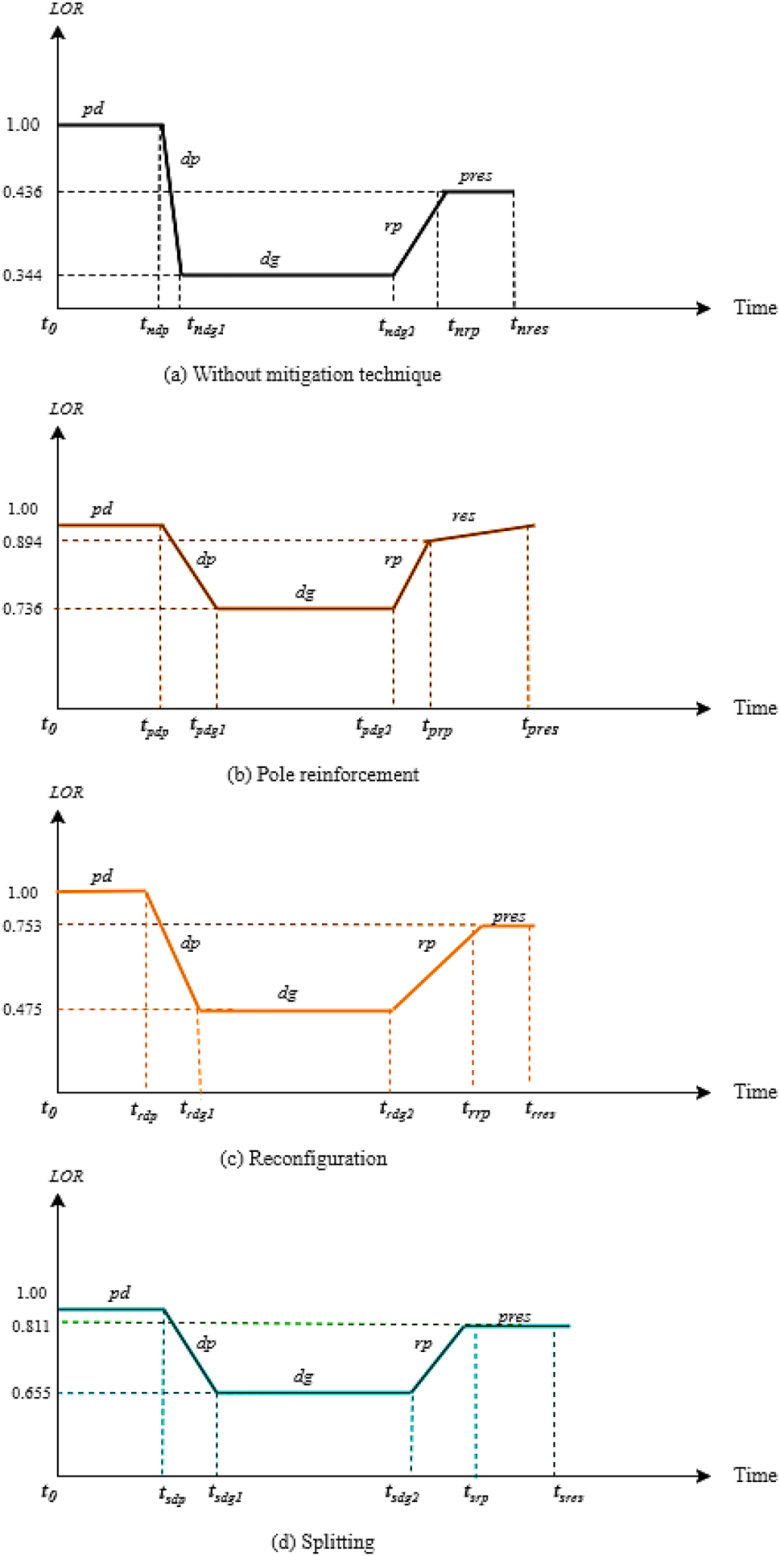
Comparative assessment of resilience trapezoid based on different mitigation techniques for cyclone prone (**a**) without any mitigation strategy (**b**) reconfiguration (**c**) splitting (**d**) pole reinforcement for region 1.

However, considering values from both Tables 4 and 5, the best strategy suited for a specific region can be decided, as shown in Fig. 5. For region 1, from Table 4 it is observed that the correlation value is highly significant for pole reinforcement (0.82 and 0.89 respectively, for intermediate and maximum trajectory case). Also, from Table 5, the restorative stage of resilience trapezoid for mitigation strategy 3 i.e., pole reinforcement shows a higher value of 0.894 as compared to reconfiguration (0.733) and splitting (0.81). Based on both the tables, the appropriate strategy of pole reinforcement can be applied to region 1. However, the initial investments are high for pole reinforcement strategy, due to the additional cost and resources required for reinforcing the poles. This is a huge initial investment, comprising labour cost, land cost, transportation, raw material, and related taxes required for this strategy. The infrastructural facility provided for it also must be robust enough to sustain the wind speed. Sometimes the temporary alternative such as using support and suspension provided to suffice the mitigation might itself be prone to damage during the wind storm. Even though pole reinforcement shows the best results but depending upon the significance of load distorted, as well as high investments, the next best alternative can be adopted. Similarly, for the rest four regions the comparison of mitigation strategies are assessed. This analysis indicates how to frame out the resilience-based planning and expansion of the system for the future.

For region 2, the resilience values for restorative states using reconfiguration, splitting, and pole reinforcement are almost the same showing 0.699, 0.616, and 0.679 respectively. This is due to the same number of nodes being taken as common for the three mitigation strategies. So, all measures lead to a similar restoration of the system and to improve the restoration of the system an immediate backup facility is required to serve the priority load locations. This makes the system available for a comparatively longer duration of time. Region 5, with average ${r}_{max}$ range of (4.96, 5.18) km and average windspeed range of (161.2, 163.3) kmph, LOR reaches 0.578, 0.501, and 0.600 for reconfiguration, splitting, and reinforcement respectively from its initial probability of 1 in normal operation. The low values are due to the high radius of cyclone trajectory which damages the major distribution lines and DG serving the priority loads. In this case also immediate DG backup facility is needed to improve the operational status of the system. This improves the resilience trapezoid nature of the region by making its restorative state value better.

For region 3 and region 4, the restorative state values for pole reinforcement are 0.856 and 0.844 respectively. These values are again better than the restorative states for reconfiguration (0.756 and 0.740) and splitting (0.776 and 0.766) for regions 3 and 4 respectively. Depending on the significance of distorted load and the total cost of pole reinforcement, the appropriate strategies can be adopted.

## Conclusion

The rapid urbanization towards SCs are driven by the core need to handle increasing power demand and addressing climatic aided power hurdles by building maximum immunity to HILF events. Distribution system being the significant framework of power system must be resilient enough to supply power to the priority consumers beyond breach. The data-driven approach using combined feature extraction property of μ-PMU and UAV, along with node prioritization using CN enabled identification of vulnerable parts within IEEE 33-bus distribution network subjected to possible cyclone trajectories. The cyclone prone zones over the world are tested and the level of resilience are quantified with resilience-based correlation considering location-oriented variables of latitude, mean elevation, and probable water level. This justified the evidence of associations between geographical and distribution system features for prioritizing vulnerable nodes and its updation using CN theory. This study helped to analyse the optimal mitigation strategy for network and meet the power need of the consumers according to expected resilience value during extreme weather scenarios. This impact on network is validated using resilience trapezoid which assesses the transitions between the different phases in which the system resides when subjected to an HILF event. Resilience based planning is thus essential to estimate the long-term impact on the system owing to varied geographical and topological characteristics. It assists in indicating the resilience-based planning and expansion of the system network, its maintenance, and a better decision making with enhanced SA.

## Abbreviations

SMs: Smart cities
HILF: High-impact low-frequency
GPS: Global positioning system
μ-PMU: Micro-phasor measurement unit
UAV: Unmanned aerial vehicle
SA: Situational awareness
DG: Distributed generation
LOS: Line-of-sight links
CN: Complex network
LOR: Level of resilience
kPCA: Kernel principal component analysis
${V}_{min}^{j}$: Minimum voltage at bus *j*
${V}_{max}^{j}$: Maximum voltage at bus *j*
${V}^{j}$: Actual voltage at bus *j*
${I}^{jk}$: Branch current between bus *j* and *k*
${C}^{j}$: Loading limit of bus *j*
$\mathit{max}O\left(\mathcal{L}\right)$: Maximum observability of network
${w}_{j}$: Relative weight of bus *j*
${\mathcal{L}}_{j}$: μ-PMUs status at bus *j*
${V}_{rated}$: Rated voltage of bus
$\varepsilon$: Tolerance limit of bus voltage
${\iota }_{ij}$: Connectivity of bus by distribution line *i* and *j*
${\Delta V}_{j}$: Change in nodal voltage at bus *j*
${\Delta I}_{jk}$: Change in branch current between buses *j* and *k*
${V}_{j}^{BE}$: Voltage at bus *j* before the event
${I}_{Jk}^{BE}$: Branch current between buses *j* and *k* before the event
${V}_{j}^{AE}$: Voltage at bus j after the event
${I}_{Jk}^{AE}$: Branch current between buses *j* and *k* after the event
*np*: Number of poles that are shaking or vibrating
$\theta$: Angle at which wind force acts on pole surface
$\varsigma$: Stress on poles due to wind force
${w}_{pole}$: Wind force on pole surface
${A}_{pole}$: Cross-section area of pole
${w}_{line}$: Wind force on overhead line
${A}_{line}$: Cross-section area of overhead line
${\mathrm{\rm M}}_{pole}$: Moment of pole due to wind force *w*
${\mathrm{\rm M}}_{g}^{\perp }$: Moment of pole due to gravitational force
${F}_{g}^{\perp }$: Force due to gravity
${d}_{g}$: Distance of pole from the ground
${\mathrm{\rm M}}_{w}^{\perp }$: Moment of pole due to wind force
${F}_{w}^{\perp }$: Wind force exerted on pole surface
${d}_{w}$: Distance of pole from the point of striking of wind force
${\mathrm{\rm M}}_{ln}^{\perp }$: Moment of overhead line due to wind force
${F}_{ln}$: Force exerted on line due to wind
${d}_{ln}$: Distance of overhead line from the point of striking of wind force
${\varsigma }_{rupture}$: Pole rupture stress
*dia*: Diameter of pole
${r}_{max}$: Radius of maximum wind speed of cyclone
${V}_{m}$: Maximum wind speed of cyclone
$\varphi$: Latitude of the considered region
*n*: Regression coefficient
$Z$: Height of the considered location
${Y}_{1}$: Exponential wind decay regression relation
${n}_{o}$: Number of observable nodes in graph v
${n}_{t}$: Total number of nodes in graph v
${\aleph }_{rank}$: Rank of critical components on a priority basis
${{\aleph }_{\mathrm{rank}}^{e}}_{new}$: Updated new rank of critical components on a priority basis
$${\mathbb{N}}_{c}$$: Node capacity
${\mathbb{N}}_{c new}$: Updated new node capacity
${D}_{n}$: Node centrality degree
${D}_{n new}$: Updated new node centrality degree
*CI*: Connectivity impact
${CI}_{new}$: Updated new connectivity impact
$\delta$: Distribution line safety margin
${l}_{c}$: Initial load of node *c*
${p}_{de}$: Probability of connection between nodes *d* and *e*
${\mathrm{\rm T}}_{a}$: Number of connected nodes after the disruptive event
${y}_{l}^{j}$: Representation of line $\mathcal{L}$ as first-order polynomial
$a$: Slope of line $\mathcal{L}$
*b*: Intercept of line $\mathcal{L}$
${\Theta }_{l}$: Slope of distribution lines
${h}_{l}^{1}$: Height of pole 1
${h}_{l}^{2}$: Height of pole 2
${\mathrm{\rm Z}}_{l}^{\mathrm{1,2}}$: Span between pole 1 and 2
$C$: General catenary curve of overhead distribution line
RNP: Resilient network parameters
MRNP: Moderately resilient network parameters
NRNP: Non-resilient network parameters
${\mathcal{R}}_{i}\left(\tau \right)$: Resilience of system at any location *i,* at time $\tau$
${LOR}_{0}$: Network’s normal level of performance
$LOR$: Network performance in the degraded state
${LOR}_{2}$: Network performance in the post-degraded state
${t}_{0}$: Initial time of pre-disturbance phase
${t}_{e}$: Time at which wind related event impacts the network
${t}_{pr}$: Time for restoration progress
${t}_{r}$: Time at which network restoration begins
${t}_{n}$: Time at which network acquires its normal state
φ: Location parameter of latitude
$\hslash$: Location parameter of mean elevation
$\varpi$: Location parameter of probable water level
$\mathcal{R}$: Expected resilience value of the network
$c{r}_{\mathcal{R},\varphi }$: Correlation of latitude with structural resilience
${cr}_{\mathcal{R},\mathcal{\hslash }}$: Correlation of elevation with structural resilience
$c{r}_{\mathcal{R},\varpi }$: Correlation of water level with structural resilience
*p*: Degree of correlation
*CC**(**∀**)*: Connected components with nodes *e* for topology mapping $\forall$
Min T: Minimum trajectory based on cyclone disturbance
Intr T: Intermediate trajectory based on cyclone disturbance
Max T: Maximum trajectory based on cyclone disturbance
${t}_{ndp}$: Disturbance progress time for without any mitigation
${t}_{ndg1}$: Degradation start time for without any mitigation
${t}_{ndg2}$: Degradation end time for without any mitigation
${t}_{nrp}$: Restoration progress time for without any mitigation
${t}_{nres}$: Recovery time for without any mitigation case
${t}_{rdp}$: Disturbance progress time for reconfiguration case
${t}_{rdg1}$: Degradation start time for reconfiguration case
${t}_{rdg2}$: Degradation end time for reconfiguration case
${t}_{rrp}$: Restoration progress time for reconfiguration case
${t}_{rres}$: Recovery time for reconfiguration case
${t}_{sdp}$: Disturbance progress time for splitting case
${t}_{sdg1}$: Degradation start time for splitting case
${t}_{sdg2}$: Degradation end time for splitting case
${t}_{srp}$: Restoration progress time for splitting case
${t}_{sres}$: Recovery time for splitting case
${t}_{pdp}$: Disturbance progress time for pole reinforcement case
${t}_{pdg1}$: Degradation start time for pole reinforcement case
${t}_{pdg2}$: Degradation end time for pole reinforcement case
${t}_{prp}$: Restoration progress time for pole reinforcement case
${t}_{pres}$: Recovery time for pole reinforcement case
*pd*: Pre-disturbance state of resilience trapezoid
*dp*: Disturbance progress rate of resilience trapezoid
*dg*: Degraded state of resilience trapezoid
*rp*: Restoration progress rate of resilience trapezoid
*pres*: Partially restored state of resilience trapezoid
*res*: Restored state of resilience trapezoid
